# Radiopharmaceutical formulation and preliminary clinical dosimetry of [^177^Lu]Lu-DOTA-MGS5 for application in peptide receptor radionuclide therapy

**DOI:** 10.1007/s00259-024-06979-1

**Published:** 2024-12-07

**Authors:** Taraneh Sadat Zavvar, Anton Amadeus Hörmann, Mark Konijnenberg, Martin Kraihammer, Christian Mair, Ariane Kronthaler, Lieke Joosten, Peter Laverman, Leonhard Gruber, Gianpaolo di Santo, Clemens Decristoforo, Irene Virgolini, Elisabeth von Guggenberg

**Affiliations:** 1 Department of Nuclear Medicine, Medical University of Innsbruck, 6020 Innsbruck, Austria; 2 Department of Radiology and Nuclear Medicine, Erasmus MC Cancer Institute, Erasmus University Medical Center, 3015 GD Rotterdam, the Netherlands; 3 Department of Medical Imaging, Nuclear Medicine, Radboud University Medical Center, 6525 GA Nijmegen, the Netherlands; 4 Department of Radiology, Medical University of Innsbruck, 6020 Innsbruck, Austria

**Keywords:** Cholecystokinin-2 receptor, Minigastrin, Peptide receptor radionuclide therapy, Lutetium-177, Theranostics, Clinical translation

## Abstract

**Purpose:**

Radiolabelled minigastrin (MG) analogues targeting the cholecystokinin-2 receptor (CCK2R) have proven to be a promising approach for peptide receptor radionuclide therapy (PRRT). In this study, we report on the radiopharmaceutical development and standardization of the preparation of [^177^Lu]Lu-DOTA-MGS5 using an automated synthesis module. Furthermore, we present the preclinical tests required to move forward towards a first therapeutic clinical trial as well as preliminary clinical dosimetry data.

**Methods:**

Five individual batches of [^177^Lu]Lu-DOTA-MGS5 were synthesized and analysed according to predefined quality control specifications. Cell-based experiments and biodistribution studies were performed to evaluate the specific receptor binding and tumour uptake of the radiopharmaceutical formulation. A preclinical dosimetry study was carried out in tumour xenografted mice and a first dosimetry study was performed in a patient with small cell lung cancer.

**Results:**

The automated cassette-based production of [^177^Lu]Lu-DOTA-MGS5 resulted in a product with high radiochemical purity of > 98% and high stability. The new radiopharmaceutical showed a favourable biodistribution profile in A431-CCK2R xenografted BALB/c nude mice. Pharmacokinetic data obtained in mice and dosimetry extrapolation demonstrated the feasibility of PRRT. In the preliminary patient-specific dosimetry study, a low risk of toxicity was shown and a mean absorbed dose of 12.5 ± 10.2 (1.2–28) Gy/GBq was calculated for delineable tumour lesions.

**Conclusion:**

The radiopharmaceutical development and the preclinical/clinical results support the initiation of a first clinical trial to evaluate the therapeutic potential of [^177^Lu]Lu-DOTA-MGS5 in PRRT.

**Supplementary Information:**

The online version contains supplementary material available at 10.1007/s00259-024-06979-1.

## Introduction

Radiotheranostic approaches, which combine diagnostic imaging and targeted therapy, have become increasingly important in nuclear medicine in recent years [1]. Peptide receptor radionuclide therapy (PRRT) is now a recognized treatment option for metastatic or inoperable neuroendocrine tumours (NET) with high overexpression of somatostatin receptors [2]. Radiolabelled minigastrin (MG) analogues targeting the cholecystokinin-2 receptor (CCK2R), overexpressed at high incidence in medullary thyroid carcinoma (MTC, 92%), small cell lung cancer (SCLC, 57%), stromal ovarian cancer (100%), astrocytomas (65%), as well as gastroenteropancreatic neuroendocrine tumours (22%), have proven to be a promising alternative approach for targeted therapy [3–5]. We have recently reported on the synthesis and preclinical characterization of a new MG analogue with improved in vivo stability and favourable tumour targeting potential. The new peptide derivative is based on DOTA-MG11 (DOTA-DGlu-Ala-Tyr-Gly-Trp-Met-Asp-Phe-NH_2_) and has two site-specific modifications in the C-terminal part of the peptide sequence. The modifications include the replacement of the terminal phenylalanine with 1-naphthylalanine (1-Nal) and the replacement of methionine with N-methylated norleucine ((N-Me)Nle), resulting in the sequence of DOTA-DGlu-Ala-Tyr-Gly-Trp-(N-Me)Nle-Asp-1-Nal-NH_2_ (DOTA-MGS5). Preliminary results demonstrated high CCK2R affinity and enhanced cellular uptake in vitro, as well as high tumour uptake and improved tumour-to-kidney ratio in xenografted mice, independent of the radiometal employed for labelling [6, 7]. With the recent successful clinical translation of [^68^Ga]Ga-DOTA-MGS5 and the initiation of a first exploratory clinical trial investigating the potential for PET/CT imaging, we were able to illustrate the capability of [^68^Ga]Ga-DOTA-MGS5 PET/CT to identify tumour sites in patients with different types of cancer [8, 9]. For further clinical implementation of the therapeutic application, we have selected the β-emitter lutetium-177 with a half-life of 6.7 days. The additional gamma ray emission allows the acquisition of quantitative SPECT images for evaluation of the targeting properties and dosimetric estimations. In this study, we report on the radiopharmaceutical development and standardization of the preparation of [^177^Lu]Lu-DOTA-MGS5 using an automated synthesis module. Furthermore, the preclinical tests required to move forward towards a first therapeutic clinical trial are presented. Cell-based in vitro experiments were carried out to evaluate saturation binding, cellular internalization and receptor-specificity. Biodistribution studies in xenografted BALB/c nude mice were performed in order to evaluate the influence of the applied amino acid substitutions, as well as of the radiopharmaceutical formulation on the tumour targeting. Furthermore, dosimetric studies in tumour xenografted mice were carried out to extrapolate radiation dose estimates for humans. Based on animal toxicity data for the unlabelled peptide conjugate generated in a GLP-conform laboratory, the initial peptide dose for clinical application was set. The first patient-individual dosimetry performed in a patient with SCLC demonstrates the feasibility of PRRT with this novel radiotherapeutic targeting CCK2R.

## Results

### Radiolabelling and quality control

**Fig. 1:**
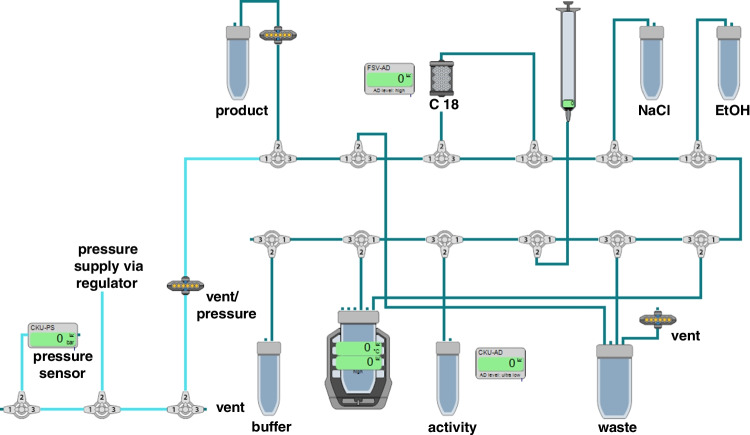
Schematic illustration of the automated synthesis process of [^177^Lu]Lu-DOTA-MGS5

The standard radiolabelling process of other in-house produced ^177^Lu-labelled radiopharmaceuticals was adjusted to the automated labelling process of [^177^Lu]Lu-DOTA-MGS5. Five separate batches of [^177^Lu]Lu-DOTA-MGS5 were evaluated. Starting activities of 3.2–10.7 GBq were reacted with 100 µg of DOTA-MGS5, resulting in a radioactivity at the end of synthesis ranging from 2.5 to 9.7 GBq. The automated synthesis process lasted around 45 min, and the volume of the final product after synthesis was 14.5 mL with a radioactivity concentration of 175–671 MBq/mL. A graphical representation of the automated synthesis is shown in Fig. 1. HPLC analysis of the final product revealed a radiochemical purity (RCP) of 98.1 ± 0.6% (*n* = 5), meeting the defined specification of ≥ 95% applied also for other radiopharmaceuticals [10]. In addition, instant thin layer chromatography (iTLC) was performed, confirming the presence of ≤ 1% of free lutetium-177 and ≤ 2% of colloidal lutetium-177. All produced batches fulfilled the defined specifications of the different parameters tested (appearance, pH, radioactivity concentration, radionuclide identity, identity, radiochemical purity, peptide content, apparent specific activity, ethanol content, bacterial endotoxins and sterility) meeting the required acceptance criteria for release. The defined specifications and results of the masterbatches are described in Supplementary Table 1 and Table 1.

**Table 1:** Quality control results of the five masterbatches of [^177^Lu]Lu-DOTA-MGS5

Parameter	Results of 5 batches
Appearance	conforms
pH	6
Volume	14.5 mL
Activity of the final product	6514 ± 2665 MBq
Radioactivity concentration	449 ± 184 MBq/mL
Radionuclide identity	conforms
Identity of [^177^Lu]Lu-DOTA-MGS5	9.51 ± 0.01 min 1.0 (RRT* versus ^nat^Lu-DOTA-MGS5)
Radiochemical purity	98.1 ± 0.6%
Free lutetium-177	0.15 ± 0.10%
Radiocolloid	0.16 ± 0.11%
Limit test for peptide content	conforms
Apparent specific activity	conforms
Ethanol content	6.9 ± 2.6% (v/v)
Bacterial Endotoxins	< 37 EU/V
Sterility	sterile

*RRT = relative retention time

**Fig. 2:**
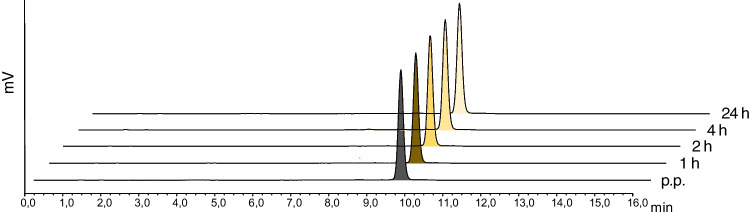
Exemplary radiochromatograms of [^177^Lu]Lu-DOTA-MGS5 p.p. and at 1, 2, 4 and 24 h p.p.

Repeated HPLC and iTLC analysis was conducted to evaluate the stability of [^177^Lu]Lu-DOTA-MGS5 stored at room temperature for the time points of 1, 2, 4 and 24 h post preparation (p.p.). A high radiochemical purity with values ≥ 95% was confirmed for [^177^Lu]Lu-DOTA-MGS5 formulated in physiological saline and < 10% ethanol, containing also sodium ascorbate (150 mg) and calcium trisodium pentatate (~ 0.7 mg). In Table 2 the stability data are summarized for the five masterbatches produced. In Fig. 2 exemplary radiochromatograms of [^177^Lu]Lu-DOTA-MGS5 at 1, 2, 4 and 24 h p.p. are depicted. For the in-house use, a shelf life of 4 h p.p. was set.

**Table 2:** Stability of [^177^Lu]Lu-DOTA-MGS5 stored at room temperature for up to 24 h p.p.

Stability testing	1 h	2 h	4 h	24 h
RCP (HPLC)	98.1 ± 0.6	97.9 ± 0.5	97.4 ± 0.6	96.6 ± 0.7
Free lutetium-177 (iTLC)	0.13 ± 0.07	0.12 ± 0.07	0.11 ± 0.05	0.12 ± 0.04
Radiocolloid (iTLC)	0.11 ± 0.05	0.09 ± 0.06	0.19 ± 0.28	0.08 ± 0.06

### Non-clinical pharmacology and toxicological data

#### Saturation binding and cell uptake studies

**Fig. 3:**
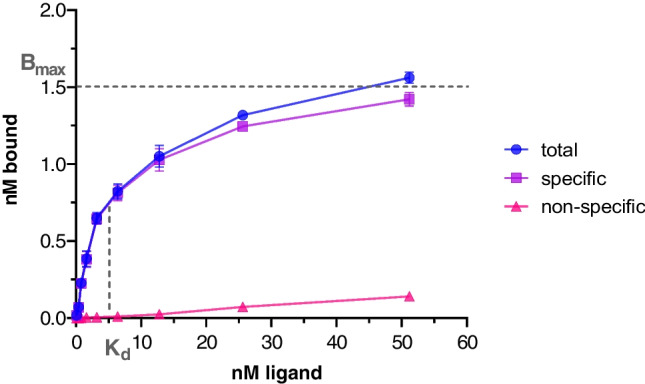
Exemplary saturation binding curve of [^177^Lu]Lu-DOTA-MGS5 on A431-CCK2R cells

In the saturation binding studies performed on A431-CCK2R cells as source for the human CCK2R, a dissociation constant (K_d_) of 5.25 ± 1.61 nM and a maximum number of binding sites (B_max_) of 1.14 ± 0.32 nM was calculated for [^177^Lu]Lu-DOTA-MGS5. In Fig. 3 an exemplary binding curve of [^177^Lu]Lu-DOTA-MGS5 is shown.

**Fig. 4:**
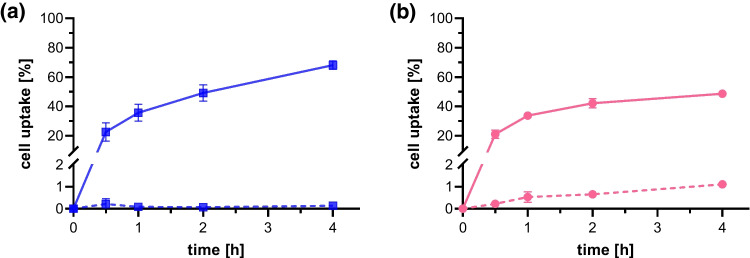
Cell uptake of [^177^Lu]Lu-DOTA-MGS5 in (**a**) A431-CCK2R, (**b**) AR42J cells. Uptake in A431-mock cells and blocking with pentagastrin in AR24J cells is additionally shown (dashed lines). The results are shown as mean ± SD based on three independent studies

In cell uptake experiments using A431-CCK2R and AR42J cells, increasing cell uptake was observed, staring from values of 22.6 ± 6.2% in A431-CCK2R cells and 21.1 ± 2.8% in AR42J cells at 30 min post incubation, the values increased to 68.0 ± 3.0% in A431-CCK2R cells and 48.6 ± 2.2% in AR42J cells for the last time point of 4 h after incubation. The non-specific uptake in A431-mock cells was < 0.5% for all studied time points. Co-incubation with 1 µM pentagastrin in AR42J cells blocked the receptor-specific uptake resulting in a non-specific internalization of ≤ 1% for all studied time points. The cell uptake of [^177^Lu]Lu-DOTA-MGS5 over time in both cell lines is shown in Fig. 4.

#### Specificity of receptor interaction

**Fig. 5:**
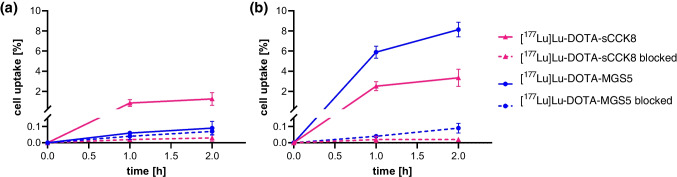
Cell uptake of [^177^Lu]Lu-DOTA-MGS5 and [^177^Lu]Lu-DOTA-sCCK8 in absence and presence of 1 µM sCCK8 for receptor blocking: (**a**) CHO-CCK1R cells, (**b**) CHO-CCK2R cells

The specificity of [^177^Lu]Lu-DOTA-MGS5 for CCK2R was confirmed using CHO-CCK1R and CHO-CCK2R cells. [^177^Lu]Lu-DOTA-sCCK8 with affinity for both CCK1R and CCK2R was used as control. A very low uptake of 0.06 ± 0.01% and 0.09 ± 0.04%, at 1 and 2 h, respectively, were observed for [^177^Lu]Lu-DOTA-MGS5 in CHO-CCK1R cells. Similar values of ~ 0.1% were observed also when co-incubating the cells with 1 µM sCCK8, confirming that no receptor-mediated uptake of [^177^Lu]Lu-DOTA-MGS5 occurred in CHO-CCKR1 cells. In CHO-CCK2R cells, the cellular uptake of 5.9 ± 0.6% and 8.1 ± 0.7% at 1 and 2 h was reduced to uptake values of less than 0.1% at 1 and 2 h by co-incubation with 1 µM sCCK8, confirming the receptor-specificity of [^177^Lu]Lu-DOTA-MGS5 for CCK2R. In comparative cell internalization studies with [^177^Lu]Lu-DOTA-sCCK8, a receptor-mediated cell uptake could be confirmed in both cell lines. Uptake values of 0.8 ± 0.3% and 1.3 ± 0.6% at 1 and 2 h were found for CHO-CCK1R cells. A somewhat higher uptake was found in CHO-CCK2R cells, with values of 2.5 ± 0.4% and 3.3 ± 0.9%, at 1 and 2 h, respectively. Co-incubation with 1 µM sCCK8 resulted in abolishment of the receptor-mediated uptake with non-specific uptake values below 0.1% in both cell lines. The cell uptake of [^177^Lu]Lu-DOTA-MGS5 and [^177^Lu]Lu-DOTA-sCCK8 in both cell lines is shown in Fig. 5.

#### Biodistribution studies in BALB/c mice

**Fig. 6:**
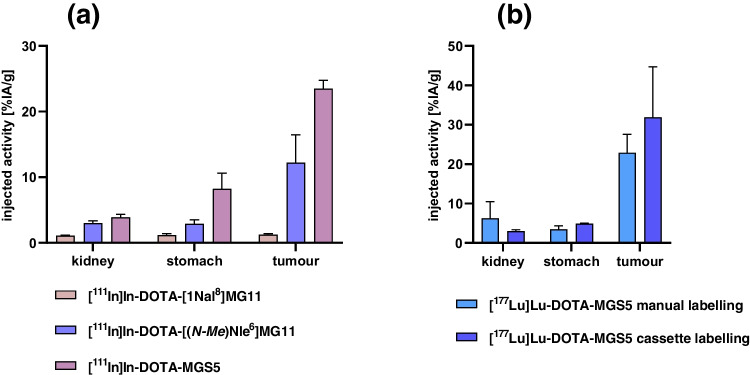
Uptake in kidney, stomach and A431-CCK2R xenografts at 4 h p.i. of (**a**): [^111^In]In-DOTA-[1Nal^8^]MG11 (*n* = 3), [^111^In]In-DOTA-[(N-Me)Nle^6^]MG11 (*n* = 3) and [^111^In]In-DOTA-MGS5 (*n* = 4) (**b**): [^177^Lu]Lu-DOTA-MGS5 prepared using manual labelling (*n* = 5) and cassette-based automated synthesis (*n* = 3)

Using peptide derivatives with single substitution with 1-Nal, single substitution with (N-Me)Nle and combined substitution with (N-Me)Nle and 1-Nal the influence of the applied amino acid substitutions on the tumour targeting properties was evaluated. To allow for a direct comparison with biodistribution data available for [^111^In]In-DOTA-MG11 without substitutions and obtained using the same conditions, this study was performed with the ^111^In-labelled peptide derivatives. The tumour uptake of the three radiopeptides in A431-CCK2R xenografts at 4 h post injection (p.i.) is shown in Fig. 6 (a) together with the uptake in kidney and stomach. A tumour uptake of 1.2 ± 0.2%IA/g was found for [^111^In]In-DOTA-[1Nal^8^]MG11, [11] whereas the uptake of [^111^In]In-DOTA-[(N-Me)Nle^6^]MG11 was 12.3 ± 4.2%IA/g. Both values were significantly lower (*P* < 0.0001 and *P* = 0.004, respectively) than the uptake of [^111^In]In-DOTA-MGS5 of 23.5 ± 1.3%IA/g previously reported [6], confirming a synergistic effect of both substitutions in improving tumour targeting. The stomach uptake of [^111^In]In-DOTA-MGS5 was also increased (8.2 ± 2.4%IA/g *versus* 1.2 ± 0.3%IA/g of [^111^In]In-DOTA-[1Nal^8^]MG11 and 2.9 ± 0.6%IA/g of [^111^In]In-DOTA-[(N-Me)Nle^6^]MG11; *P* = 0.004 and *P* = 0.014, respectively). Also the kidney uptake of [^111^In]In-DOTA-MGS5 (3.9 ± 0.5%IA/g) was higher when compared to [^111^In]In-DOTA-[1Nal^8^]MG11 (1.1 ± 0.1%IA/g, *P* = 0.0001) and [^111^In]In-DOTA-[(N-Me)Phe^6^]MG11 (3.0 ± 0.3%IA/g; *P* = 0.039). Still, tumour-to-organ ratios of [^111^In]In-DOTA-MGS5 with values of 3.1 ± 1.1 for stomach and 6.1 ± 0.6 for kidney were favourable.

Using the same mouse tumour model, the biodistribution of [^177^Lu]Lu-DOTA-MGS5 prepared using the cassette-based synthesis process was compared with standard manual labelling. The results obtained from this study are shown in Fig. 6 and allow the comparison of the tumour uptake with other MG analogues recently studied in clinical trials [12]. A comparable uptake in A431-CCK2R xenografts was observed, with values of 22.9 ± 4.7%IA/g found for [^177^Lu]Lu-DOTA-MGS5 (tumour weight: 436 ± 200 mg; *n* = 5) prepared by manual labelling and values of 31.9 ± 12.8%IA/g for [^177^Lu]Lu-DOTA-MGS5 (tumour weight: 185 ± 103 mg; *n* = 3) prepared using the automated synthesis process, at 4 h after injection. A higher variability in tumour uptake was found for A431-CCK2R xenografts of BALB/c nude mice injected with [^177^Lu]Lu-DOTA-MGS5 prepared using a cassette-based synthesis process, however no statistical significance was found between the two groups for tumour xenografts as well as all other tissues analysed.

#### Preclinical pharmacokinetics and dosimetry

**Fig. 7:**
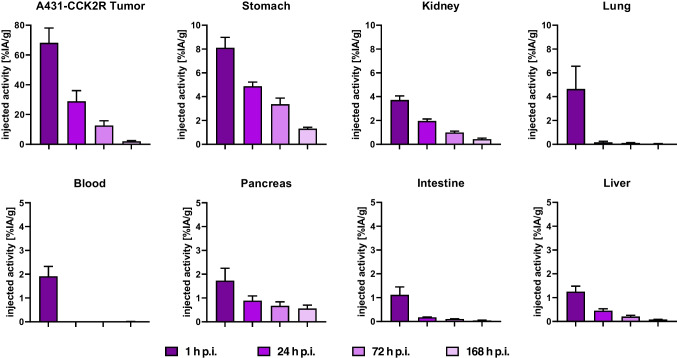
Time-dependent distribution of [^177^Lu]Lu-DOTA-MGS5 in A431-CCK2R xenografted BALB/c nude mice for selected tissues over time (*n* = 5, for each time point)

In the dosimetry study performed for [^177^Lu]Lu-DOTA-MGS5 intravenously injected into A431-CCK2R xenografted female BALB/c nude mice, adequate tumour targeting was confirmed. A very high accumulation of radioactivity in A431-CCK2R xenografts and good retention of the radioactivity over time was observed. The initial tumour uptake of 68.1 ± 10.0%IA/g at 1 h p.i., decreased to values of 28.9 ± 7.2%IA/g and 12.6 ± 3.3%IA/g, at 24 h and 72 h p.i., respectively. The radioactivity remained detectable in the tumour also at 7 days p.i. (2.0 ± 0.5%IA/g). A rapid washout of the radioactivity was observed from the blood pool, with most organs showing relatively low radioactivity accumulation. The radioactivity in the blood pool decreased from 1.9 ± 0.4%IA/g at 1 h to 0.01 ± 0.002%IA/g at 24 h p.i., resulting in undetectable levels throughout the further course of the study. A low uptake of 3.7 ± 0.4%IA/g was observed in the kidneys 1 h p.i., decreasing to 2.0 ± 0.2%IA/g at 24 h and levels of < 1%IA/g for all later time points studied. The initial uptake in the receptor-positive stomach was 8.1 ± 0.9%IA/g at 1 h p.i., and gradually declined to 4.9 ± 0.4%IA/g, 3.4 ± 0.5%IA/g and 1.3 ± 0.1%IA/g at 24 h, 72 h, and 168 h p.i., respectively. The radioactivity accumulation and washout over time is shown in Fig. 7 for selected organs. In Supplementary Table 2, the uptake values over time are summarized for all dissected tissues and organs analysed. The tumour-to-organ ratios for the most relevant organs are shown in Supplementary Table 3. The absorbed dose found in different tissues and organs are shown in Supplementary Table 4.

**Fig. 8:**
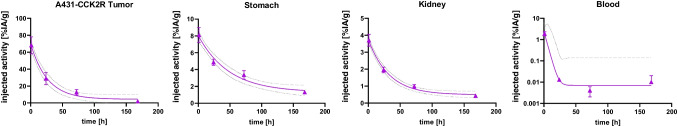
Time activity curves in A431-CCK2R xenografts, blood, stomach and kidney p.i. of [^177^Lu]Lu-DOTA-MGS5 in A431-CCK2R-xenografted BALB/c nude mice. Single-exponential curve fits are shown with 95% confidence intervals

The time-activity curves were fitted to the biodistribution data with single-exponential functions with R^2^ > 0.7 (Fig. 8). The clearance from the tumour proceeded with a biological half-life of 18.2 h (12.3–27.1 h; 95% confidence interval). The blood clearance proceeded with a half-life of 2.8 h (0–7.6 h; 95% confidence interval).

Based on dosimetry extrapolation from mice to humans, the expected absorbed doses of [^177^Lu]Lu-DOTA-MGS5 in human organs were calculated and are shown in Table 3. An estimated radiation dose for stomach of 0.045 mGy/MBq for males and 0.051 mGy/MBq for females was calculated, taking into account the stomach wall as the source of radiation. A predicted radiation dose of 0.018 mGy/MBq for males and 0.022 mGy/MBq for females was found for kidneys. Assuming an injected radioactivity of 7,400 MBq for each treatment cycle with [^177^Lu]Lu-DOTA-MGS5, a stomach dose of ~ 354 mGy and kidney dose of ~ 148 mGy can be expected, resulting in a cumulative dose of 1.4 Gy for stomach and 0.6 Gy for kidneys after 4 therapeutic cycles.

**Table 3:** Expected absorbed dose of [^177^Lu]Lu-DOTA-MGS5 in humans

Target organ	Absorbed dose per injected activity [mGy/MBq]	
	Male	Female
Colon wall	0.003	0.004
Small intestine	0.006	0.007
Stomach wall	0.045	0.051
Kidneys	0.018	0.022
Heart wall	0.001	0.002
Liver	0.005	0.007
Lungs	0.003	0.004
Pancreas	0.016	0.019
Red marrow	0.001	0.001
Spleen	0.005	0.006
Urinary bladder wall	0.001	0.001
	**Effective dose (mSv/MBq)**	
**Whole Body**	0.007	0.008

#### Toxicity study in rats

In a previously performed extended singe-dose toxicity study [13], the intravenous administration of DOTA-MGS5 at the three different dose levels of 0.1, 0.5, and 2.5 mg/kg body weight was well tolerated in Wistar rats. A NOAEL (no observed adverse effect level) of 0.4 mg/kg body weight was established for humans. For the first exploratory clinical trial with [^177^Lu]Lu-DOTA-MGS5, a starting dose of 100 µg was set. This dose remains well below the 1/100 NOAEL of 4 µg/kg. The performed study justifies the use of [^177^Lu]Lu-DOTA-MGS5 in a microdose trial without therapeutic intent, investigating the safety of administration and dosimetry aspects (microdose approach 1). When following the microdose approach 2, a maximum of five administrations with each dose ≤ 100 µg and a total cumulative dose of ≤ 500 µg would be acceptable. The performed non-clinical evaluation also forms the basis for a therapeutic trial using [^177^Lu]Lu-DOTA-MGS5 in patients with advanced cancer and limited therapeutic options [14].

#### First patient-specific clinical dosimetry

**Fig. 9:**
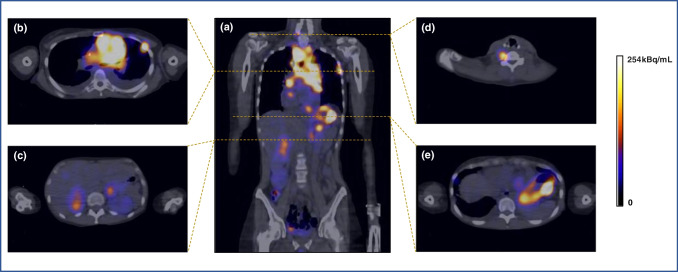
[^177^Lu]Lu-DOTA-MGS5 SPECT/CT scan at 24 h p.i. (**a**) fused coronal view of the full body scan. Dashed areas indicate the level of the axial slices at (**b**) left-central primary tumour and cervical/mediastinal lesions, as well as a left subpleural lesion, (**c**) both adrenal glands lesions, (**d**) right cervical vertebral metastasis and (**e**) soft tissue lesion in the gastric curvature together with physiological uptake in the gastric wall. The scale is set at 30% of the maximum activity concentration of 845 kBq/ml

Planar and quantitative SPECT/CT imaging performed after administration of 1.5 GBq [^177^Lu]Lu-DOTA-MGS5 in a patient with extensive disease small cell lung cancer (ED-SCLC) revealed high uptake in tumour lesions. In Fig. 9 a fused coronal view of the full body SPECT/CT scan as well as axial slices at 24 h p.i. are given. Physiological accumulation was observed mainly in the urinary system and in the gastrointestinal tract (receptor-mediated uptake in stomach; intestinal activity at later time points). For all tumour lesions, clear uptake was confirmed, predominantly in the left-central primary tumour and cervical/mediastinal lesions, both adrenal glands metastases, as well as different bone and soft tissue lesions. The preliminary patient-specific dosimetry study showed a low risk of renal and bone marrow toxicity (absorbed doses 0.28 Gy/GBq and 0.022 Gy/GBq, respectively) and receptor-mediated uptake in stomach (0.42 Gy/GBq). In the dosimetric calculations performed on five well delineable lesions an absorbed dose of 12.5 ± 10.2 (1.2–28) Gy/GBq was found confirming the feasibility of PRRT.

## Discussion

The aim of this study was to standardise the preparation of [^177^Lu]Lu-DOTA-MGS5 and to comprehensively assess the pharmaceutical and non-clinical characteristics of [^177^Lu]Lu-DOTA-MGS5 for its possible clinical application. The initial clinical outcomes utilizing [^68^Ga]Ga-DOTA-MGS5 as an imaging probe revealed that this PET tracer exhibits the capacity to identify local recurrence and metastases in patients with CCK2R-positive tumours [8, 9].

The result of this work demonstrates the therapeutic potential of [^177^Lu]Lu-DOTA-MGS5 and provides the non-clinical analysis that can be used as a basis for a first clinical trial application. The MG analogue, DOTA-MGS5, used in this study, includes two minor amino acid modifications applied in the receptor-specific sequence. By comparing the biodistribtuion profile of ^111^In-labelled peptide derivatives with single or combined substitution, we could show the synergistic effect of the two modifications in enhancing the targeting properties of DOTA-MGS5. In A431-CCK2R xenografts, [^111^In]In-DOTA-MGS5 showed a tumour uptake of ~ 24% IA/g which was ~ two fold higher compared to [^111^In]In-DOTA-[(N-Me)Nle^6^]MG11 and ~ 20-fold higher compared to [^111^In]In-DOTA-[1Nal^8^]MG11 with single substitution, respectively. Also, the tumour-to-kidney and tumour-to-stomach ratio of [^111^In]In-DOTA-MGS5 were favourable when compared with the peptide derivatives with single substitution. This study was carried out using the ^111^In-labelled peptide analogues to allow for comparison with data of other ^111^In-labelled MG analogues previously studied [15] and recently used in clinical trials [16]. A similar improvement of tumour uptake so far was achieved only by co-administration of phosphoramidon, an inhibitor of neutral endopeptidase (NEP) improving the bioavailability of the radiopeptide. This approach allowed to increase the tumour uptake of [^111^In]In-DOTA-MG11 from values of ~ 2–3 to 10–16% ID/g [15, 17]. The tumour uptake of [^111^In]In-DOTA-MGS5 in A431-CCK2R xenografts resulted to be four times higher also when compared to MG analogues recently used in clinical trials. The uptake of [^111^In]In-DOTA-MGS5 in A431-CCK2R xenografts resulted to be more than doubled when compared to [^111^In]In-DOTA-PP-F11 (< 10%IA/g, 4 h p.i.) and also the tumour-to-organ ratios were improved [18]. In agreement with previous reports [6, 7, 26], also for [^177^Lu]Lu-DOTA-MGS5 a high tumour uptake of ~ 23%ID/g was confirmed, which is clearly superior when compared to ^177^Lu-labelled PP-F11 and PP-F11N both showing a tumour uptake of ~ 7%ID/g in the same tumour model, which could only be further increased to ~ 9% ID/g by co-administration of phosphoramidon [12].

The five batches of [^177^Lu]Lu-DOTA-MGS5 produced using an automated cassette-based module all fulfilled the defined product specifications and a high radiochemical purity and stability was confirmed up to 24h after preparation. In the clinical setting, the implementation of an automated cassette-based module enables the reproducible production of the radiolabelled peptide with high yields and pharmaceutical quality [19].

In the non-clinical testing, a high receptor affinity with a K_d_ value of 5.25 ± 1.61 nM was observed for [^177^Lu]Lu-DOTA-MGS5 on A431-CCK2R cells. Using the same cell line, for [^111^In]In-DOTA-PP-F11 and [^177^Lu]Lu-DOTA-PP-F11, with the amino acid sequence of DOTA-(DGlu)_6_-Ala-Tyr-Gly-Trp-Met-Asp-Phe-NH_2_, a K_d_ value of 15.9 ± 2.2 and 41.7 ± 9.7, respectively, was reported [12, 20]. For [^177^Lu]Lu-DOTA-PP-F11N, with the amino acid sequence of DOTA-(DGlu)_6_-Ala-Tyr-Gly-Trp-Nle-Asp-Phe-NH_2_, a similar value of 50.0 ± 7.3 nM was found [12]. The B_max_ of 1.14 ± 0.32 nM found for [^177^Lu]Lu-DOTA-MGS5 in this study is in a comparable range when compared to [^111^In]In-DOTA-PP-F11 (1.21 ± 0.06), and somewhat higher when compared to [^177^Lu]Lu-DOTA-PP-F11 (0.65 ± 0.06) and [^177^Lu]Lu-DOTA-PP-F11N (0.54 ± 0.10) [12, 20].

The specificity of DOTA-MGS5 for CCK2R was confirmed using CHO cells expressing either CCK1R or CCK2R. The CCK-family peptides contain a Tyr moiety preceding the C-terminal receptor binding motif. The Tyr residue is involved in determining the receptor specificity [21]. sCCK8, a cholecystokinin-8 analogue with sulphated Tyr residue, exhibits a strong affinity to both CCK1R and CCK2R and was used as a control peptide [22]. [^177^Lu]Lu-DOTA-sCCK8, showed receptor-specific uptake in both cell lines, whereas [^177^Lu]Lu-DOTA-MGS5 only showed a specific uptake in CCK2R expressing cells, whereas no receptor-mediated uptake was observed in CCK1R positive cells.

In the biodistribution study comparing [^177^Lu]Lu-DOTA-MGS5 using either standard manual labelling or the cassette-based synthesis process no significant differences in the overall biodistribution and tumour targeting properties were observed, demonstrating the suitability of the standardised process for clinical use. A somewhat higher tumour uptake combined with a higher standard deviation due to the lower tumour weight and lower number of animals was found for A431-CCK2R xenografts of BALB/c nude mice injected with [^177^Lu]Lu-DOTA-MGS5 prepared by a cassette-based synthesis process. On the basis of a 7-day biodistribution study in A431-CCK2R xenografted BALB/c nude mice, dosimetric calculations were performed and extrapolated to the absorbed dose in humans. The highest absorbed dose was found for the stomach wall (0.05 ~ mGy/MBq) which is related to the physiological expression of CCK2R in the stomach. Besides stomach, the kidneys as the main route of excretion represent a dose-limiting organ. An expected absorbed dose of ~ 0.02 mGy/MBq was calculated for this organ. Clinical dosimetry was performed only for [^177^Lu]Lu-PP-F11N in six patients with advanced MTC [23]. The median absorbed tumour dose achieved was 0.88 Gy/GBq (range: 0.63–3.59 Gy/GBq), which is lower than the absorbed dose of 2.0 Gy/GBq delivered by [^177^Lu]Lu-DOTA-TATE targeting somatostatin receptors and used for PRRT in neuroendocrine tumours [23, 24]. The clinical dosimetry data with [^177^Lu]Lu-PP-F11N suggest the stomach as a dose limiting organ with a median absorbed dose of 0.42 (range: 0.13–1.66) Gy/GBq for the stomach wall and a median tumour-to-stomach ratio of 3.34 (range: 0.54–9.45) Gy/GBq. The median absorbed dose to kidney was 0.11 (range: 0.05–0.15) Gy/GBq with a median tumour-to-kidney ratio of 11.6 (range:6.5–27.6) [23]. A recent study assessed the effect of NEP inhibitors in combination with [^177^Lu]Lu-PP-F11N in eight patients with advanced MTC. Premedication with Entresto (sacubitril/valsartan) improved the absorbed tumour dose (median: 0.74 Gy/GBq vs. 0.28 Gy/GBq). However, no significant improvement in tumour-to-organ ratios was observed [25]. In the first patient-specific dosimetry study performed with [^177^Lu]Lu-DOTA-MGS5 in a patient with ED-SCLC, a mean tumour dose of 12.5 ± 10.2 (1.2–28) Gy/GBq was found in the delineable lesions. The absorbed dose to the stomach wall was 0.42 Gy/GBq achieving a mean tumour-to-stomach dose of 30. The dose delivered to kidney was 0.28 Gy/GBq, with a mean tumour-to-kidney ratio of 45. The preliminary clinical data in a patient with SCLC demonstrate the feasibility of using [^177^Lu]Lu-DOTA-MGS5 for PRRT in patients with CCK2R-expressing tumours.

## Conclusion

The performed preclinical studies demonstrate that [^177^Lu]Lu-DOTA-MGS5 possesses promising targeting characteristics, along with high tumour uptake and favourable tumour retention over time. Furthermore, the standardised production of [^177^Lu]Lu-DOTA-MGS5 using an automated cassette-based module and the fulfilment of specific quality control criteria was validated. Radiation-associated risk assessment using dosimetry estimates from animal data provided a justifiable risk to patients. The first dosimetry study in a patient with SCLC confirmed an enhanced tumour dose and favourable tumour-to-organ ratios. These findings serve as the basis for the initiation of a first clinical trial to assess the safety, tolerability, and therapeutic potential of [^177^Lu]Lu-DOTA-MGS5 in PRRT.

## Materials and methods

### Validation of the radiosynthesis and quality control

Five individual batches of [^177^Lu]Lu-DOTA-MGS5 were synthesized using a Modular-Lab PharmTracer® (Eckert & Ziegler Eurotrope GmbH, Berlin, Germany) and analysed using predefined quality control specifications (Supplementary Table 1). Details of the automated synthesis method are described in Supplementary Materials. Repeated HPLC and iTLC analyses up to 24 h p.p. were carried out to evaluate the stability of the final product.

### Non-clinical pharmacology and toxicology data

#### Radiolabelling

Radiolabelling with indium-111 or lutetium-177 for non-clinical testing was accomplished using 2–10 µg of DOTA conjugate, ~ 50–400 MBq of [^111^In]InCl_3_ or [^177^Lu]LuCl_3_ solution, and a > 1.2-fold volume of a 0.4 M sodium acetate/0.24 M gentisic acid solution (pH 5) with a radioactivity concentration of ~ 0.3–3 MBq/µL. The reaction was carried out in a low protein binding tube (Eppendorf AG, Hamburg, Germany) incubated at ~ 90 °C for 20 min. A SepPak® tLight C18 cartridge (Waters, Milford, MA, USA) was used to purify reaction solutions before use in biodistribution studies. A detailed description of the radiolabelling and purification process can be found elsewhere [7, 26].

#### Cell lines, saturation binding studies and cell uptake studies

The Supplementary Materials provide a detailed description of the different cell lines (A431-CCK2R/mock, AR42J, CHO-CCK1R and CHO-CCK2R) as well as of the cell based experiments.

#### Comparative biodistribution studies in BALB/c mice

Biodistribution studies were performed in female athymic BALB/c nude mice (Charles River Laboratories, Sulzfeld, Germany) with A431-CCK2R xenografts induced and animals treated as described previously [7]. The study was approved by the Austrian Ministry of Science (2022–0.351.553). Groups of three animals were intravenously injected with ~ 300 kBq [^111^In]In-DOTA-[(N-Me)Nle^6^]MG11, [^111^In]In-DOTA-[1Nal^8^]MG11, [^111^In]In-DOTA-MGS5, and ~ 800 kBq [^177^Lu]Lu-DOTA-MGS5 corresponding to 20 pmol peptide. [^177^Lu]Lu-DOTA-MGS5 produced by the automated module was used in the biodistribution study in order to compare the equivalence of the biodistribution profile with [^177^Lu]Lu-DOTA-MGS5 produced by manual labelling. Statistical analysis was performed on the data sets using the unpaired two-tailed t-test with a significance level of 0.05.

#### Preclinical pharmacokinetics and dosimetry

A biodistribution study up to 7 days p.i. was performed in female athymic BALB/c nude mice (Charles River Laboratories, Sulzfeld, Germany) in order to evaluate the tissue distribution and tumour uptake of [^177^Lu]Lu-DOTA-MGS5 over time. The study was approved by the Dutch animal ethics committee (CCD) of the Radboud University and the Nijmegen Medical Centre animal ethics committee (RUDEC) (2020–0007–020). Mice were inoculated with A431-CCK2R cells (2 × 10^6^ in 200 μL DMEM medium) at an age of 8–10 weeks. After the formation of tumour xenografts (after dissection a tumour size of 0.32 ± 0.14 g was determined, *n* = 20), mice were randomly divided into four groups (*n* = 5/group) and injected via a lateral tail vein with [^177^Lu]Lu-DOTA-MGS5 (~ 1 MBq, 20 pmol of peptide) diluted in 200 µL of PBS/0.5% BSA (*n* = 20). At different time points (1 h, 24 h, 72 h, and 168 h p.i.), animals were euthanized by CO_2_/O_2_-asphyxiation, a blood sample was drawn immediately, and various tissues (lung, heart, femur, muscle, spleen, intestine, liver, kidney, stomach, pancreas, A431-CCK2R tumour) were dissected. Samples were collected, weighted and measured in a gamma counter together with a 1% standard of the injected dose. Results were expressed as percentage of injected activity per gram of tissue (%IA/g), and tumour-to-organ activity ratios were calculated for selected organs. The biodistribution data were used to calculate the kinetics in mouse organs and A431-CCK2R xenografts. A detailed description of the dosimetry calculations and extrapolation from mice to humans can be found in the Supplementary Materials.

#### Toxicity study in rats

The first clinical dose for the therapeutic application was established based on a previous extended singe-dose toxicity study performed on the chemical precursor DOTA-MGS5 in a GLP-compliant laboratory (BSL BIOSERVICE Scientific Laboratories Munich GmbH, Planegg/Munich, Germany) [13]. According to applicable guidelines [27–29] a targeted programme of non-clinical testing was followed. The safety was assessed in a single species and no repeated dose study was performed, as complete clearance between administrations is expected. The study was conducted in male Wister rats and included three dose groups and one control group. The histopathological examination was focused on the main target organs. In addition, a dosimetry study was performed in A431-CCK2R xenografted BALB/c nude mice to demonstrate adequate tumour targeting.

### First human dosimetry

A 40 year-old female patient with ED-SCLC was intravenously administered with 1.5 GBq [^177^Lu]Lu-DOTA-MGS5 (≤ 100 µg, 14.5 mL) allowing for dosimetric calculations. SPECT/CT scans were performed with a NM CT 870 DR SPECT/CT system (GE Healthcare, USA). Planar whole body scans as well as two-field (thorax and abdomen) SPECT/CT scans were carried out at approximately 0.5, 4, 24, 72 and 96 h after administration. The details of the dosimetric calculations can be found in the Supplementary Materials. The examination was performed on a named-patient basis, and written informed consent was obtained from the patient in accordance with the Declaration of Helsinki.

## Supplementary Information

Below is the link to the electronic supplementary material.

Supplementary file1 (DOCX 53 KB)

## Data Availability

The datasets generated during and/or analysed during the current study are available from the corresponding author on reasonable request. For open access purposes, the author has applied a CC BY public copyright license to any author accepted manuscript version arising from this submission.
