# Supplementary material for: Radiopharmaceutical formulation and preliminary clinical dosimetry of [177Lu]Lu-DOTA-MGS5 for application in peptide receptor radionuclide therapy
Source: Eur J Nucl Med Mol Imaging. 2024 Dec 7;52(4):1321–31. doi: 10.1007/s00259-024-06979-1 (PMC11839890; doi:10.1007/s00259-024-06979-1)
Supplement: Supplementary file 1 — Supplementary file1 (DOCX 53 KB) [file 259_2024_6979_MOESM1_ESM.docx]

**Supplementary Materials:**

**Radiopharmaceutical formulation and preliminary clinical dosimetry of [^177^Lu]Lu-DOTA-MGS5 for application in peptide receptor radionuclide therapy**

Taraneh Sadat Zavvar^1^, Anton Amadeus Hörmann^1^, Mark W Konijnenberg^2^, Martin Kraihammer^1^, Christian Mair^1^, Ariane Kronthaler^1^, Lieke Joosten^3^, Peter Laverman^3^, Leonhard Gruber^4^, Gianpaolo di Santo^1^, Clemens Decristoforo^1^, Irene Virgolini^1^, Elisabeth von Guggenberg^1^*

^1^ Department of Nuclear Medicine, Medical University of Innsbruck, 6020 Innsbruck, Austria

^2^ Department of Radiology and Nuclear Medicine, Erasmus MC Cancer Institute, Erasmus University Medical Centre, 3015 GD Rotterdam, the Netherlands

^3^ Department of Medical Imaging, Nuclear Medicine, Radboud University Medical Centre, 6525 GA Nijmegen, the Netherlands

^4^ Department of Radiology, Medical University Innsbruck, 6020 Innsbruck, Austria

* Correspondence: [elisabeth.von-guggenberg@i-med.ac.at](mailto:elisabeth.von-guggenberg@i-med.ac.at)

**Keywords:** cholecystokinin-2 receptor; minigastrin; peptide receptor radionuclide therapy; lutetium-177, theranostics, clinical translation

**Supplementary Materials and Methods**

**Validation of the Radiosynthesis and Quality Control**

Five individual batches of [^177^Lu]Lu-DOTA-MGS5 were synthesized at the Radiopharmaceutical Laboratory (clean room class C) of the Department of Nuclear Medicine of the Medical University of Innsbruck. A Modular-Lab PharmTracer® (Eckert & Ziegler Eurotrope GmbH, Berlin, Germany) in combination with a disposable sterile cassette suitable for the preparation of therapeutic radiopharmaceuticals (C4-Y90-00 or Lu177-00#01, Eckert & Ziegler Eurotrope GmbH, Berlin, Germany) placed in a hot cell (class B) was used for the synthesis. The Modular-Lab PharmTracer software (Modular-Lab SoftPLC 6.2; Eckert & Ziegler) was used to run the production of [^177^Lu]Lu-DOTA-MGS5, which provided a graphical overview of each process step and the synthesis progression. The cassette was tested for pressure leakage before starting the synthesis to check the integrity of the tubes and valves. A 10-mL syringe pump operated by the software ensured the transport of all liquids during the synthesis. The reagents used in the synthesis are described in the following section and were loaded into the system using the disposable material from the accessory kit of the cassette. A 250 µg vial of DOTA-MGS5 GMP grade (piCHEM, Raaba-Grambach, Austria) was dissolved in 20-50% ethanol (1 µg/µL) using 95% ethanol (alcohol concentrate 95%, 20 mL ampule, B. Braun Melsungen, Germany) and aqua bidest. (10 mL ampule, Fresenius Kabi, Graz, Austria). An aliquot of 100 µL of this solution was transferred to the reaction vial of the cassette. A 50 mg ascorbate buffer kit (ASC-01, Polatom, Otwock, Poland) was dissolved in 1.5 mL of aqua bidest and was transferred to the buffer vial of the cassette. The following additives were added to the product vial using a 0.22 µm sterile filter (Millex GV, SLGV013SL, Merck Millipore, Co. Cork, Ireland): 20-fold molar excess over the peptide of DTPA – using a solution prepared from calcium trisodium pentetate (Ditripentat-Heyl®, 5 ml ampule, Heyl, Berlin, Germany) and physiological saline (50 mL vial, Fresenius Kabi, Graz, Austria) - and 1 mL of an injectable ascorbic acid solution (Vitamin C, 150 mg/mL Pascoe, Giessen, Germany). A 20 mL vial of 80% ethanol – prepared from 95% ethanol and aqua bidest – and a 50 mL vial of physiological saline (Fresenius Kabi, Graz, Austria) were further connected to the cassette. Before starting the synthesis, the vial containing the ^177^Lu-chloride solution (non-carrier added LuCl_3_ in 0.04 N HCl (ITM Medical Isotopes, Garching/Munich, Germany or Isotopia Nuclear Medicine, Park Tetach Tikva, Isreal) was intruded into the hot cell in a lead-shielded container and connected to the activity line of the cassette. The product line was connected to the 0.22 µm sterile filter (Millex-GV, SLGV033RS, Merck Millipore, Co. Cork, Ireland) placed at the product vial. The synthesis process started with the activation of the SepPak cartridge (C18 light, WAT023501 Waters, Milford, MA, USA) with 5 mL of 80 % ethanol and 5 mL of physiological saline, followed by the transfer of the ^177^Lu-chloride solution and the ascorbic acid buffer to the reaction vial. The reaction mixture was heated for 20 min at 85°C and purified on the SepPak cartridge. The final injectable solution was formulated by eluting [^177^Lu]Lu-DOTA-MGS5 from the cartridge with 1 mL of 80% ethanol through the 0.22 µm sterile filter into the product vial, followed by dilution with 12 mL of physiological saline to obtain a final volume of 14.5 mL. An integrity test of the sterile filter was performed by checking the pressure leakage of the product line and sterile filter. Every synthesis step was documented in the synthesis report.

For the final product, the volume of 14.5 mL was confirmed using a graduated vial. The appearance was assessed by visual inspection and the pH of the solution was tested using a pH indicator strip (1.09526.0003, Universal indicator, Merck). The radioactivity was measured in the calibrator and the radioactivity concentration as well as the apparent specific activity was calculated based on the radioactivity at the end of synthesis and the peptide amount. The radionuclide identity of lutetium-177 was verified by means of gamma-ray spectroscopy and by checking for the prominent gamma photons of lutetium with an energy of 208 and 113 keV [1]. The identity of the final product was confirmed by high-performance liquid chromatography (HPLC) analysis using DOTA-MGS5 labelled with the stable isotope of lutetium (Lu-DOTA-MGS5) as a reference standard. For the validation of the synthesis process and the analysis of the acceptance criteria of the masterbatches, an UltiMate 3000 chromatography system (Dionex, Germering, Germany) was used. The system consists of a HPLC pump, an autosampler, a variable UV-detector (UV-VIS at λ = 280 nm), and a radiodetector (GabiStar, Raytest, Straubenhardt, Germany) together with an ACE 3 µm C18 column, 150×3 mm (ACE-111-1503, Advanced Chromatography Technologies Ltd., Aberdeen, Scotland). As mobile phases 0.1% TFA in water (A) and 0.1% TFA in acetonitrile (B) were used together with a flow rate of 0.6 mL/min and the following gradient: 0–2 min 28% B, 2–12 min 28–55% B, 12–12.1 min 55–28% B, 12.1–16 min 28% B. Chromeleon Dionex Software (Version 7.2.9) was used as analysing software. The peak area associated to [^177^Lu]Lu-DOTA-MGS5 relative to the total radioactivity in the chromatogram was identified using a relative retention time (RRT) of 0.9-1.1 of the reference standard and the percentage of this peak was used to determine the RCP. In addition, iTLC using iTLC-SG glass microfiber chromatography paper impregnated with silica gel, 9×1 cm (SGI0001, Agilent, Waldbronn, Germany), was performed for the evaluation of the presence of free lutetium-177 (0.1 M sodium citrate pH 5, Rf >0.8) and radiocolloid (1:1 mixture of 1 M ammonium acetate and methanol, Rf <0.2). A Scan-RAM radio-TLC scanner with a PS Plastic/PMT detector (LabLogic Systems, Sheffield, UK) was used to determine the percentage of impurities.

Repeated HPLC and iTLC analysis up to 24h after preparation was carried out to evaluate the stability of the final product.

A limit test for the peptide amount was performed using a calibration curve generated with DOTA-MGS5 at a UV wavelength of 280 nm.

Bacterial endotoxin testing was performed immediately after synthesis. An Endosafe PTS reader and PTS-20F Limulus Amebocyte Lysate (LAL) cartridges (Charles River Laboratories, Charleston, SC, USA) were used for this purpose [2]. The ethanol content (gas chromatography) and the sterility (in accordance with Pharm. Eur. by an accredited laboratory) were determined after a decay time corresponding to 10 times the half-life of lutetium-177. The specifications for the final product were set based on the experience in the in-house production of other radiotherapeutics and Ph. Eur. monographs available for other radiopharmaceuticals [3].

**Cell Lines**

The A431 human epidermoid carcinoma cell line stably transfected with the plasmid pCR3.1 containing the complete human CCK2R coding sequence and the same cell line transfected with the empty vector alone were originally provided by Dr. Luigi Aloj [4]. AR42J rat pancreatic cancer cells expressing rat CCK2R were obtained from the European Collection of Authenticated Cell Cultures (ECACC, Salisbury, UK). Chinese hamster ovary (CHO) cells that were stably transfected with cDNA encoding for either CCK1R or CCK2R were kindly provided by Dr. Peter Laverman. A431 and CHO cells were grown in Dulbecco's modified Eagle's medium (DMEM) supplemented with 10% (v/v) foetal bovine serum and 5 mL of a 100x penicillin-streptomycin-glutamine mixture. AR42J cells were grown in RPMI-1640 medium supplemented with 10% (v/v) foetal bovine serum and 5 mL of a 100x penicillin-streptomycin-glutamine mixture. Cells were cultured in a humidified atmosphere containing 5% CO_2_ at 37°C. Cells were passaged three times per week with 10x 2.5% trypsin-EDTA solution at a 1:2-1:3 ratio. All media and supplements were purchased from Sigma-Aldrich (Darmstadt, Germany) or Invitrogen Corporation (Lofer, Austria).

**Saturation Binding Studies**

The saturation binding assay was performed on A431-CCK2R cells plated 2 days prior to the assay at a density of 1×10^6^ cells per well in 6-well plates. For the assessment of binding affinity, cells were incubated with serial dilutions of [^177^Lu]Lu-DOTA-MGS5 in culture medium at 4°C for 30 min to 1h with concentrations ranging from 0.1 to 200 nM. In order to assess non-specific binding, the serial dilutions of [^177^Lu]Lu-DOTA-MGS5 were additionally co-incubated with 1 µM of unlabelled peptide. Before recovering the cell-associated radioactivity from the wells by using 1 M NaOH (two times), cells were washed twice with ice-cold phosphate-buffered saline (PBS). The radioactivity was then counted using a gamma counter (2480 Wizard2 3”, PerkinElmer Life Sciences and Analytical Instruments, formerly Wallac Oy, Turku, Finland) and non-specific binding was subtracted. For normalization to the number of cells, cells from three different wells were trypsinized and counted by Beckman Coulter Vi-CELL AS cell viability analyser (Beckman Coulter, Fullerton, CA, USA). Three independent assays were performed in triplicate. Binding curves were analysed using GraphPad Prism version 10.1.2 for Windows (GraphPad Software, Boston, Massachusetts USA).

**Cell Uptake Studies**

The cell uptake of [^177^Lu]Lu-DOTA-MGS5 was studied in the A431-CCK2R/mock and AR42J cell lines over time using a previously published method [5]. Cells were incubated with [^177^Lu]Lu-DOTA-MGS5 over a time of 0.5-4h using a peptide concentration of 0.4 nM per well. For the A431 cell line, the mock cells without the overexpression of CCK2R were used to determine the non-specific uptake. For AR42J cells, a blocking study with 1 µM of pentagastrin was performed. The percentage of radioactivity in relation to the total activity added to each well (% of cell uptake) was calculated.

The specificity of [^177^Lu]Lu-DOTA-MGS5 for CCK2R was studied in CHO cells stably transfected with either CCK1R or CCK2R. ^177^Lu-labelled DOTA-sCCK8 (Asp-Tyr(SO_3_H)-Met-Gly-Trp-Met-Asp-Phe-NH_2_) binding to both CCK1R and CCK2R was studied for comparison. The cells were seeded at a density of 1×10^6^ in 6-well plates 48h prior to the assay. On the day of the experiment, the medium was discarded and the cells were incubated with the radioconjugated peptides (~50,000 cpm; 0.4 nM) in DMEM medium containing 1% FBS at 37 °C. The cell uptake was analysed in the absence or presence of 1 µM sCCK8 (Eurogentec, Ougrée, Belgium) as a blocking solution. The same protocol used for A431 and AR42J cells was used to determine the cell uptake. Each experiment was performed three times in triplicate and the mean value and standard deviation for all the experiments was calculated.

**Preclinical Pharmacokinetics and Dosimetry**

A one-compartment distribution model was used to characterize the time-activity curves, which were fitted with a single-exponential function implementing Graphpad Prism software. The time-integrated activity concentration coefficients [TIAC] were determined by integrating the exponential curves folded with the ^177^Lu decay function with a half-life of 6.7 d. Animal dosimetry was performed using the RADAR mouse phantom [6]. The mouse phantom organ weights were used to translate the time-integrated activity concentration coefficients [TIAC] into the regular TIAC. The absorbed dose per given activity was calculated using the MIRD equation (medical internal radiation dose) and the S values obtained from the 25 g RADAR mouse phantom.

$$D=\left[ \mathrm{TIAC}\left( \mathrm{organ} \right) \right] \times m organ \times S (organ\leftarrow organ)$$

Extrapolation of the mouse-based dosimetry to human absorbed doses was done using the equation presented below. In addition, time scaling was applied using a second equation to account for the more rapid kinetics in mice compared to humans [7, 8]:

$\% IA per organ in humans=\left[ \%(\frac{\mathrm{IA}}{g})mice \times mass of mice \left( \mathrm{kg} \right) \right]\times( \frac{mass of human organ (g)}{total body mass of human (kg)}$ )

$\mathrm{time} \mathrm{in} \mathrm{humans}=\mathrm{time} \mathrm{in} \mathrm{mice} \times[ \frac{\mathrm{mass} \mathrm{of} \mathrm{human} (kg)}{\mathrm{mass} \mathrm{of} \mathrm{mice} (kg)}$ ] $\frac{1}{4}$

The TIACs generated via the extrapolation method were used as input in the IDAC-dose dosimetry software [9]. Organ absorbed doses were computed for adult females and males.

**First human Dosimetry**

For patient dosimetry calculations, the sensitivity of the gamma camera was calibrated according to the recommended workflow by GE Healthcare (General Electric Co. DICOM Conformance Statement: NM General Purpose 600/800 Series. Rev. 15. GE Healthcare. 2018), using a petri dish filled with a known activity of Lu-177 resulting in a calibration factor of 5.2 cps/MBq. An IEC-NEMA phantom (Data Spectrum corporation, USA) with fillable spheres of different sizes as well as a Jaszczak phantom (Data Spectrum corporation, USA) with a sphere of 60 mm diameter were used to account for the partial volume effect and subsequently determine the respective recovery curve. A diagnostic CT scan as well as a previous [^68^Ga]Ga-DOTA-MGS5 PET/CT were used for organ and tumour segmentation in Affinity 3.0 (Hermes Medical Solutions). Volumes of all other organs were estimated by means of OLINDA and subsequently BMI-corrected. Time-activity curves were fitted by three-exponential functions for organs at risk (kidneys, stomach wall) and lesions, as well as two-exponential curves for the whole body and remainder body to obtain time-integrated activity. The respective VOIs were transferred to the planar whole-body images in the case of the remainder body. Subsequently, organ doses were evaluated with commercial OLINDA (Hermes Medical Solutions) software. Tumour doses were calculated by means of OLINDA’s sphere model by approximating them with spheres of the same volume and corrected for partial volume effect. Blood sampling and measurements were done according to the EANM bone marrow and whole-body dosimetry guideline to calculate bone marrow absorbed dose [10].

**Supplementary Table 1.** Acceptance criteria of [^177^Lu]Lu-DOTA-MGS5

| Parameter | Method | Limits |
| --- | --- | --- |
| Appearance | Visual inspection | Clear, colourless solution, free of visible particles |
| pH | Indicator strip | 5-7 |
| Volume | Graduated vial | 10-20 mL |
| Activity of the final product | MBq | >1000 MBq |
| Radioactivity Concentration | MBq/mL | <700 |
| Radionuclide identity | Gamma-ray spectrometry  (113 and 208 kev) | conforms |
| Identity of  [^177^Lu]Lu-DOTA-MGS5 | HPLC Rt  (RRT versus ^nat^Lu-DOTA-MGS5) | 0.9-1.1 |
| Radiochemical purity | RCP (HPLC) | ≥95% |
| Free lutetium-177 | TLC (0.1 sodium citrate pH 5)  Rf 0.8-1.0 | <1% |
| Radiocolloid | TLC (1 M ammonium acetate/methanol; 1/1); Rf 0-0.3 | <2% |
| Limit test for peptide content | HPLC (UV) | ≤100 µg/V |
| Apparent specific activity | MBq/µg | >20 MBq/µg |
| Ethanol content | Gas chromatography (v/v) | ≤10% (v/v) |
| Bacterial Endotoxins | LAL test | <175 EU/V |
| Sterility | Ph. Eur. | sterile |

**Supplementary Table 2**. Uptake values of [^177^Lu]Lu-DOTA-MGS5 in A431-CCK2R-xenografted BALB/c nude mice for up to 7 days after injection (n = 5, for each time point).

| Organs | 1h p.i.  [%IA/g] | 24h p.i.  [%IA/g] | 72h p.i.  [%IA/g] | 168h p.i.  [%IA/g] |
| --- | --- | --- | --- | --- |
| Blood | 1.9±0.4 | 0.013±0.002 | 0.004±0.002 | 0.01±0.01 |
| Lung | 4.6±1.9 | 0.2±0.1 | 0.10±0.04 | 0.04±0.01 |
| Heart | 0.8±0.2 | 0.05±0.01 | 0.026±0.005 | 0.018±0.004 |
| Femur | 0.3±0.1 | 0.052±0.008 | 0.032±0.007 | 0.06±0.02 |
| Muscle | 0.3±0.2 | 0.04±0.01 | 0.010±0.003 | 0.018±0.005 |
| Spleen | 0.6±0.1 | 0.21±0.05 | 0.14±0.05 | 0.09±0.01 |
| Intestine | 1.1±0.3 | 0.17±0.02 | 0.10±0.01 | 0.04±0.01 |
| Liver | 1.3±0.2 | 0.5±0.1 | 0.2±0.1 | 0.08±0.01 |
| Kidney | 3.7±0.4 | 2.0±0.2 | 1.0±0.1 | 0.4±0.1 |
| Stomach | 8.1±0.9 | 4.9±0.4 | 3.4±0.5 | 1.3±0.1 |
| Pancreas | 1.7±0.5 | 0.9±0.2 | 0.7±0.2 | 0.6±0.1 |
| A431-CCK2R | 68.1±10.0 | 28.9±7.2 | 12.6±0.3 | 2.0±0.5 |

**Supplementary Table 3.** Tumour-to-organ ratios of [^177^Lu]Lu-DOTA-MGS5 in A431-CCK2R-xenografted BALB/c nude mice for up to 168h after injection (n = 5 mice per time point).

| Time point p.i. | 1h | 24h | 72h | 168h |
| --- | --- | --- | --- | --- |
| Tumour-to-blood | 36.4±5.8 | 2283.0±307.5 | 3366.3±1588.0 | 329.2±200.0 |
| Tumour-to-stomach | 8.5±1.4 | 6.0±1.7 | 3.8±0.9 | 1.5±0.4 |
| Tumour-to-kidney | 18.4±1.8 | 14.8±3.2 | 12.7±2.6 | 4.5±0.7 |

**Supplementary Table 4**. Absorbed dose per injected activity for [^177^Lu]Lu-DOTA-MGS5 in A431-CCK2R-xenografted BALB/c nude mice

| Target organ | Absorbed dose per injected activity [mGy/MBq] |
| --- | --- |
| Large intestine | 53.7 |
| Small intestine | 51.1 |
| Stomach wall | 388.6 |
| Kidneys | 179.3 |
| Liver | 52.4 |
| Lungs | 44.3 |
| Pancreas | 140.0 |
| Bone | 19.5 |
| Spleen | 40.4 |
| Tumour | 2122.7 |
|  | **Effective dose (mSv/MBq)** |
| Whole Body | 52.4 |

**References:**

1. European Directorate for the Quality of Medicines and Health Care. Lutetium (^177^Lu) solution for radiolabelling. European Pharmacopoeia EDQM. 2020:1218-9.

2. European Directorate for the Quality of Medicines and Health Care. Bacterial endotoxins. European Pharmacopoeia EDQM. 2020:209-13.

3. Pharmeuropa. Lutetium (^177^Lu) Zadavotide Guraxetan Injection. 2023;(35.2 edition):04/2023:3170

4. Aloj L, Caracò C, Panico M, Zannetti A, Del Vecchio S, Tesauro D, et al. In vitro and in vivo evaluation of 111In-DTPAGlu-G-CCK8 for cholecystokinin-B receptor imaging. J Nucl Med. 2004;45:485-94.

5. Zavvar TS, Hörmann AA, Klingler M, Summer D, Rangger C, Desrues L, et al. Effects of side chain and peptide bond modifications on the targeting properties of stabilized minigastrin analogs. Pharmaceuticals. 2023;16:278.

6. Keenan MA, Stabin MG, Segars WP, Fernald MJ. RADAR realistic animal model series for dose assessment. J Nucl Med. 2010;51:471-6.

7. Fani M, Weingaertner V, Kolenc Peitl P, Mansi R, Gaonkar RH, Garnuszek P, et al. Selection of the first 99mTc-labelled somatostatin receptor subtype 2 antagonist for clinical translation—Preclinical assessment of two optimized candidates. Pharmaceuticals. 2020;14:19.

8. INTERNATIONAL ATOMIC ENERGY AGENCY, Guidance for Preclinical Studies with Radiopharmaceuticals. IAEA Radioisotopes and Radiopharmaceuticals Series No 8. 2023:69.

9. Andersson M, Johansson L, Eckerman K, Mattsson S. IDAC-Dose 2.1, an internal dosimetry program for diagnostic nuclear medicine based on the ICRP adult reference voxel phantoms. EJNMMI Res. 2017;7:1-10.

10. Hindorf C, Glatting G, Chiesa C, Lindén O, Flux G. EANM Dosimetry Committee guidelines for bone marrow and whole-body dosimetry. Eur J Nucl Med Mol Imaging. 2010;37:1238-50.
